# Supplementary figures and images for: Do sex hormones confound or mediate the effect of chronotype on breast and prostate cancer? A Mendelian randomization study
Source: PLoS Genet. 2022 Jan 21;18(1):e1009887. doi: 10.1371/journal.pgen.1009887 (PMC8809575; doi:10.1371/journal.pgen.1009887)

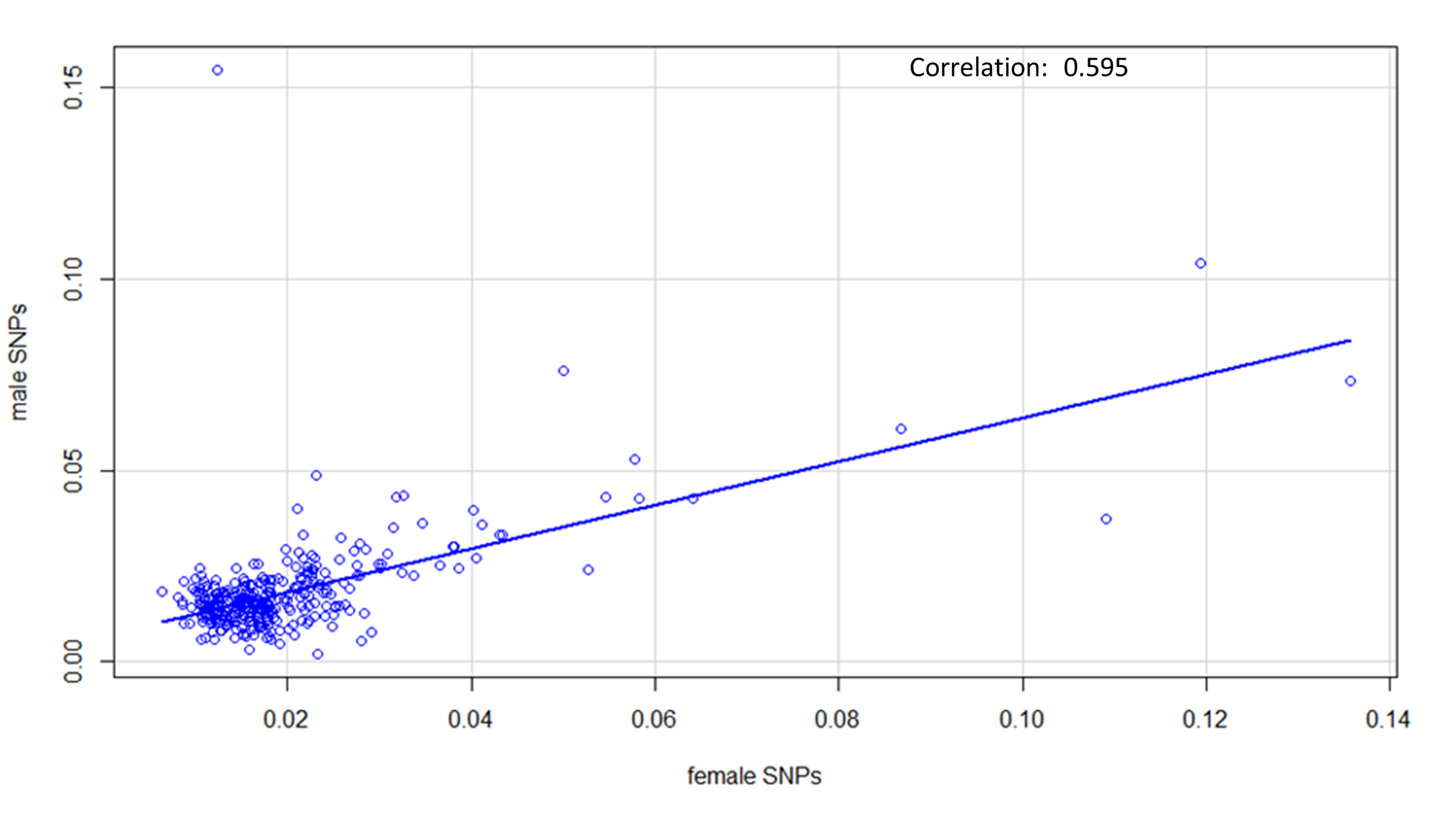

Supplement: S1 Fig — (TIF) [file pgen.1009887.s003.tif]

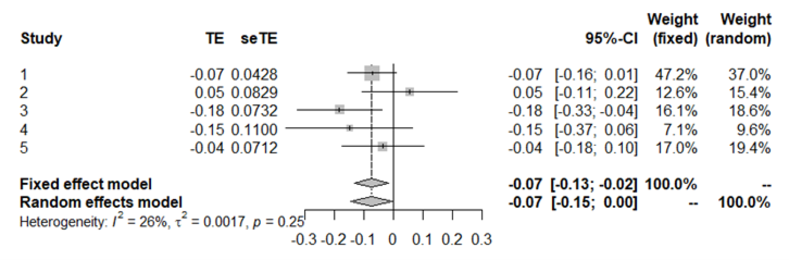

Supplement: S2 Fig — Study numbers = 1. Luminal A (ER+/PR+, HER2-); 2. Luminal B (ER+/PR+/-, HER2+); 3. Luminal B (ER+/PR+/-, HER2-, Ki 67 > 14%); 4. HER2 (ER-, PR-, HER2+); and 5. Triple Negative (ER-, PR-, HER2-). (TIF) [file pgen.1009887.s004.tif]

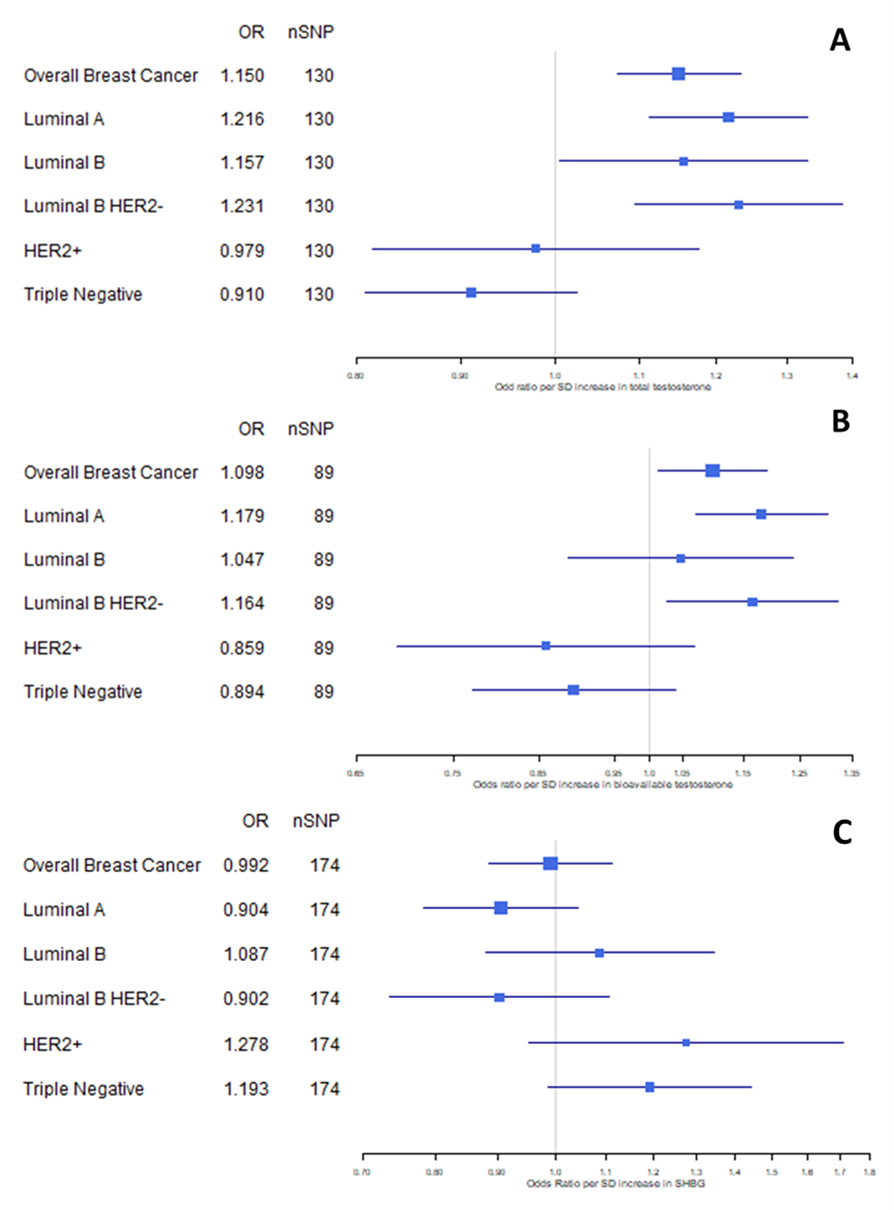

Supplement: S3 Fig — Forest plot of uvMR results for A) total testosterone, B) bioavailable testosterone and C) SHBG effects on breast cancer subtypes. (TIF) [file pgen.1009887.s005.tif]

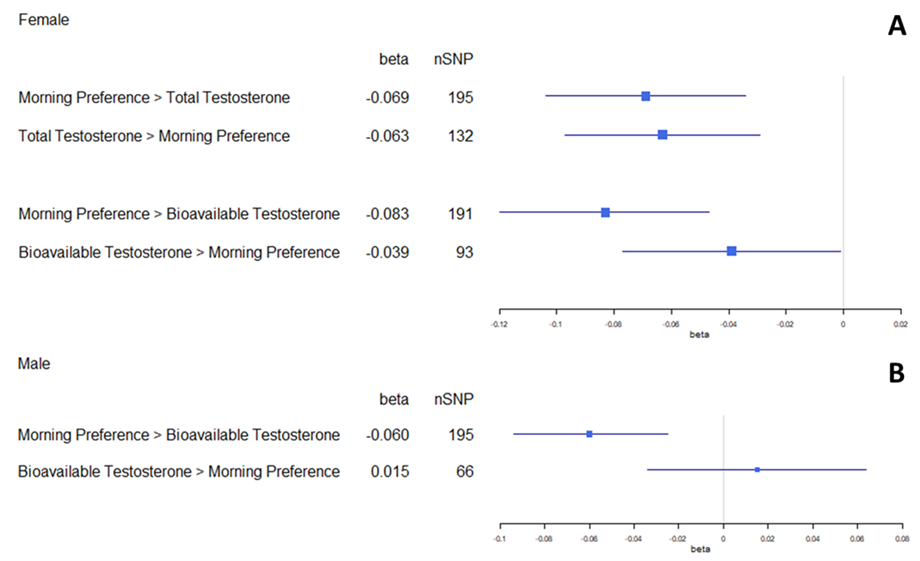

Supplement: S4 Fig — Forest plot of split sample bdMR results for morning preference and sex hormones in female (A) and male (B) datasets. All instruments in these analyses have been subjected to Steiger-filtering. (TIF) [file pgen.1009887.s006.tif]

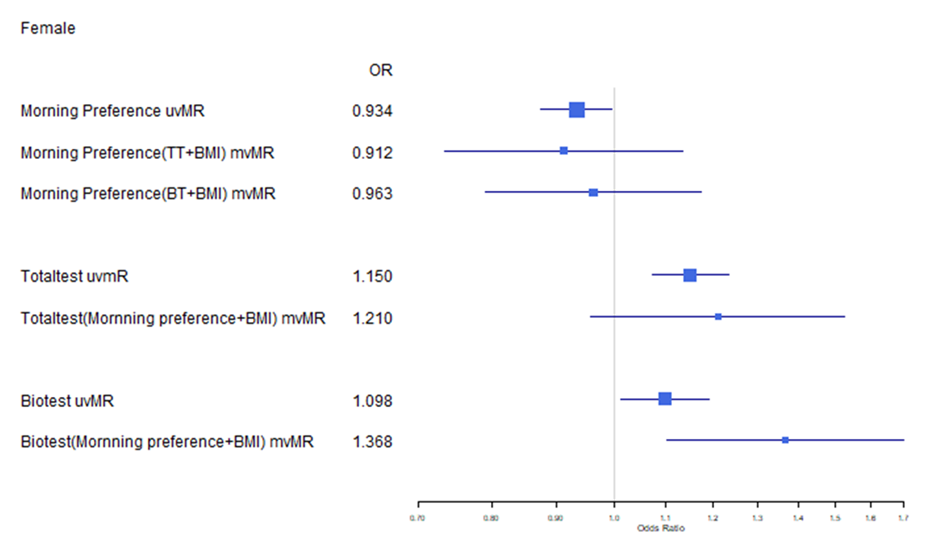

Supplement: S5 Fig — uvMR results provided for comparison. (TIF) [file pgen.1009887.s007.tif]

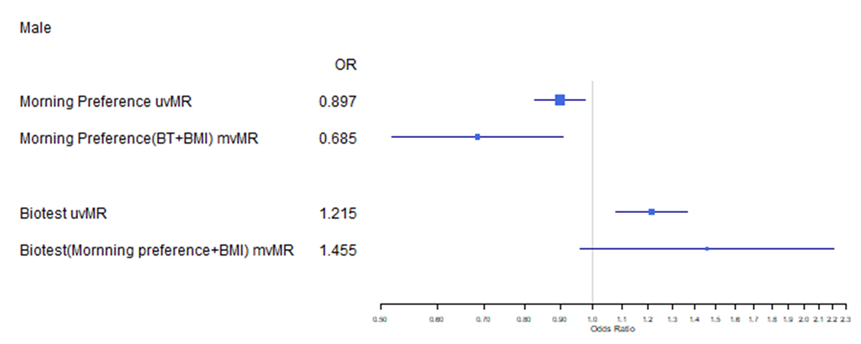

Supplement: S6 Fig — uvMR results provided for comparison. (TIF) [file pgen.1009887.s008.tif]
